# Estrogen Receptor Alpha (ESR1)-Dependent Regulation of the Mouse Oviductal Transcriptome

**DOI:** 10.1371/journal.pone.0147685

**Published:** 2016-01-25

**Authors:** Katheryn L. Cerny, Rosanne A. C. Ribeiro, Myoungkun Jeoung, CheMyong Ko, Phillip J. Bridges

**Affiliations:** 1 Department of Animal and Food Sciences, University of Kentucky, Lexington, KY 40546, United States of America; 2 Department of Clinical Sciences, University of Kentucky, Lexington, KY 40536, United States of America; 3 Department of Comparative Biosciences, University of Illinois at Urbana-Champaign, Urbana, IL 61802, United States of America; University of Quebec at Trois-Rivieres, CANADA

## Abstract

Estrogen receptor-α (ESR1) is an important transcriptional regulator in the mammalian oviduct, however ESR1-dependent regulation of the transcriptome of this organ is not well defined, especially at the genomic level. The objective of this study was therefore to investigate estradiol- and ESR1-dependent regulation of the transcriptome of the oviduct using transgenic mice, both with (ESR1KO) and without (wild-type, WT) a global deletion of ESR1. Oviducts were collected from ESR1KO and WT littermates at 23 days of age, or ESR1KO and WT mice were treated with 5 IU PMSG to stimulate follicular development and the production of ovarian estradiol, and the oviducts collected 48 h later. RNA extracted from whole oviducts was hybridized to Affymetrix Genechip Mouse Genome 430–2.0 arrays (n = 3 arrays per genotype and treatment) or reverse transcribed to cDNA for analysis of the expression of selected mRNAs by real-time PCR. Following microarray analysis, a statistical two-way ANOVA and pairwise comparison (LSD test) revealed 2428 differentially expressed transcripts (DEG’s, P < 0.01). Genotype affected the expression of 2215 genes, treatment (PMSG) affected the expression of 465 genes, and genotype x treatment affected the expression of 438 genes. With the goal of determining estradiol/ESR1-regulated function, gene ontology (GO) and bioinformatic pathway analyses were performed on DEG’s in the oviducts of PMSG-treated ESR1KO versus PMSG-treated WT mice. Significantly enriched GO molecular function categories included binding and catalytic activity. Significantly enriched GO cellular component categories indicated the extracellular region. Significantly enriched GO biological process categories involved a single organism, modulation of a measurable attribute and developmental processes. Bioinformatic analysis revealed ESR1-regulation of the immune response within the oviduct as the primary canonical pathway. In summary, a transcriptomal profile of estradiol- and ESR1-regulated gene expression and related bioinformatic analysis is presented to increase our understanding of how estradiol/ESR1 affects function of the oviduct, and to identify genes that may be proven as important regulators of fertility in the future.

## Introduction

Gamete storage and maturation, fertilization, and early embryonic development occur in the oviduct. Ovarian-derived estradiol is a known regulator of a oviductal function, modulating contraction of its smooth musculature [1,2] and secretion by its epithelial cells [3,4], with the transcription factors estrogen receptor-α (ESR1) and estrogen receptor-β (ESR2) both expressed within this organ. ESR1 has been localized to ciliated and secretory epithelial cells, stromal cells, as well as smooth muscle cells of the oviduct [5,6,7], whereas ESR2 appears to be largely confined to ciliated epithelial cells [6]. Immunoreactivity of both receptor subtypes to the nucleus, cytoplasm and plasma membranes [8] indicative of both genomic and non-genomic responses. Estradiol also acts as a regulator of ESR1 protein expression [5,6,7], estradiol and ESR1 therefore acting as important transcriptional regulators where hormone bound receptors target the estrogen responsive element (ERE) on the promoter region of their target genes to either enhance or repress transcription [9,10].

Genome-wide reports on estradiol/ESR1-dependent regulation of oviductal function are lacking. Our objective was therefore to identify estradiol/ESR1-dependent transcriptomal changes in the oviduct using a mouse model that is deficient in ESR1 expression (ESR1KO), testing the hypothesis that estradiol, acting through ESR1, affects the expression of mRNAs within this organ. Immature mice were utilized to circumvent the reproductive phenotype observed in ESR1KO mice observed following puberty, where females develop cystic ovaries and dysfunction of the hypothalamic-pituitary axis due to elevated concentrations of circulating estradiol [11,12]. Immature mice (ESR1KO and WT) were left untreated, or were treated with PMSG to stimulate follicular development and the production of estradiol. Microarray-based transcriptional profiling and bioinformatic analyses was therefore performed using oviducts collected from mice bearing a global deletion of ESR1 and their wild-type (WT) littermates, both before and after PMSG-induced production of ovarian estradiol.

To provide the reader with full access to the transcriptomal dataset, the raw data (*.cel files) plus the GCRMA-normalized and log_2_ transformed transcript data (Park Genomics Suite [13]), have been deposited into the Gene Expression Omnibus (National Center for Biotechnology Information [14]) as accession number GSE72614 (http://www.ncbi.nlm.nih.gov/geo).

## Materials and Methods

### Animals and Tissue Collection

All animal procedures were approved by the University of Kentucky Institutional Animal Care and Use Committee. Mice with a global deletion of ESR1 (ESR1KO) on a C57BL/6 background were generated as previously described [15,16]. Briefly, two transgenic mouse lines were used; male ESR1^flox/flox^ were bred with female Zp3^cre^ to produce a line expressing Cre recombinase in the oocyte. The F1 heterozygotes (ESR1^flox/+^Zp3^cre^) were bred with ESR1^flox/flox^ resulting in ESR1^flox/flox^Zp3^cre^ mice, where females produce oocytes that are ESR1^-^. ESR1^flox/flox^Zp3^cre^ females were then bred with ESR1^flox/-^ males to produce ESR1KO progeny (ESR1^-/-^ and ESR1^-/-^ ZP3^Cre^) or sibling controls (ESR1^flox/-^ and ESR1^flox/-^ ZP3^Cre^). Genomic DNA was extracted from ear punches using the Easy DNA kit (Invitrogen, Carlsbad CA) to confirm genotypes, as previously described [16]. Whole oviducts were collected for extraction of RNA from immature female mice (ESR1KO and WT) killed at 23 days of age, or ESR1KO and WT mice treated i.p. with 5 IU PMSG at 23 days of age and killed 48 h later.

### RNA Extraction

Oviducts were pooled from 3–4 mice per treatment group and genotype and total RNA was extracted using TRIzol Reagent (Invitrogen, Carlsbad, CA) and purified through RNeasy columns (Qiagen, Valencia, CA), as described before [15,17]. RNA was analyzed for quality and quantified by spectrophotometry using an Eppendorf BioPhotometer Plus (Eppendorf, Germany) as well as by visual distinction of 18S and 28S rRNA bands after ethidium bromide staining in an agarose gel. Spectrophotometry revealed a mean 260/280 ratio of 1.75 ± 0.10 for all samples. Aliquots of the same total RNA were used for both microarray and real-time reverse-transcription PCR (real-time RT-PCR).

### Microarray Hybridization

A total of 12 microarray hybridizations were performed using the Affymetrix Genechip Mouse Genome 430–2.0 arrays (GeneChip; Affymetrix, Inc., Santa Clara, CA) according to the manufacturer’s instructions at the University of Kentucky Microarray Core Facility, as described before [15,17,18]. Three replicates using different mice were generated for each treatment group.

**Fig 1:**
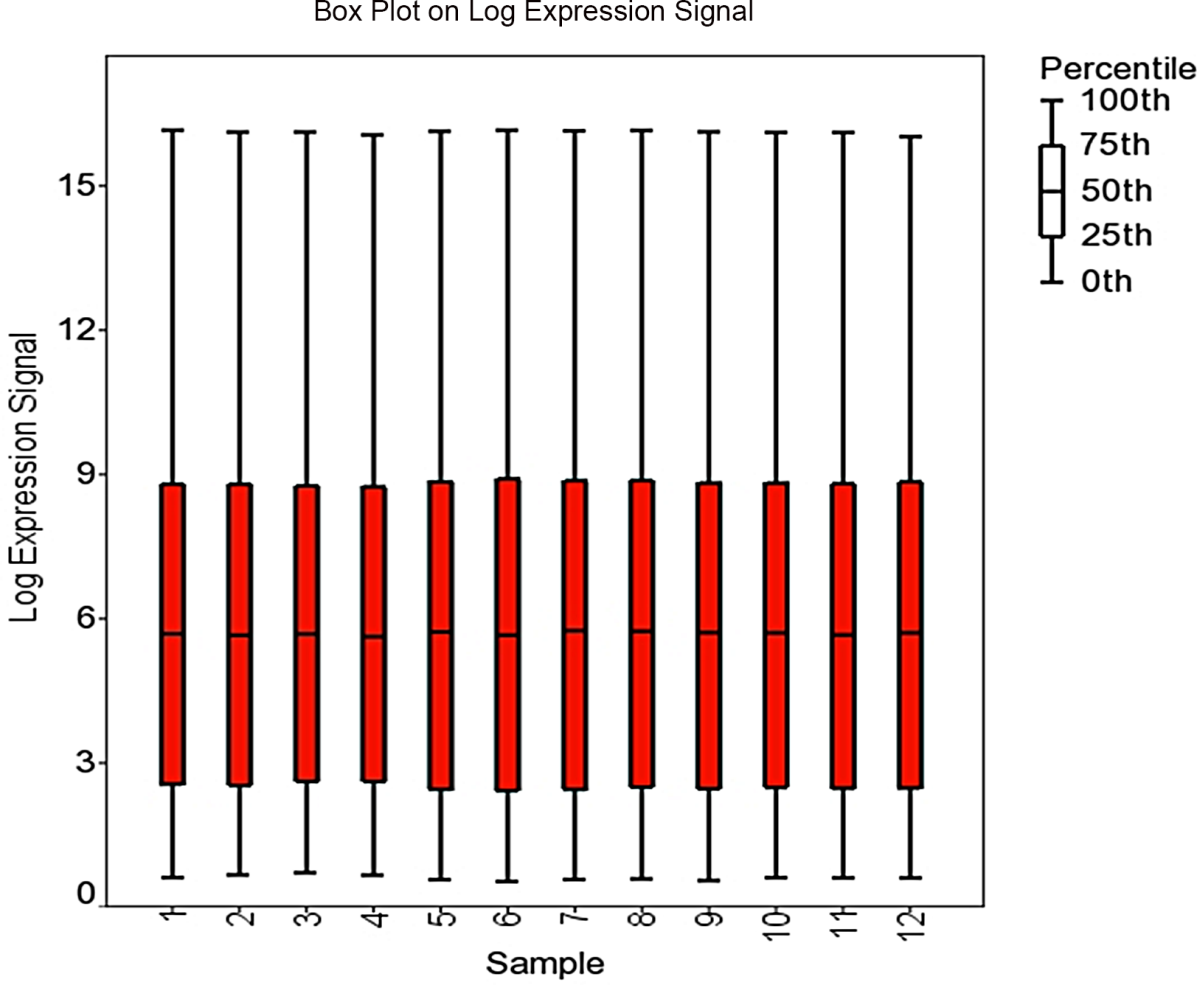
Box plot of the log_2_ expression signal for each microarray chip.

**Fig 2:**
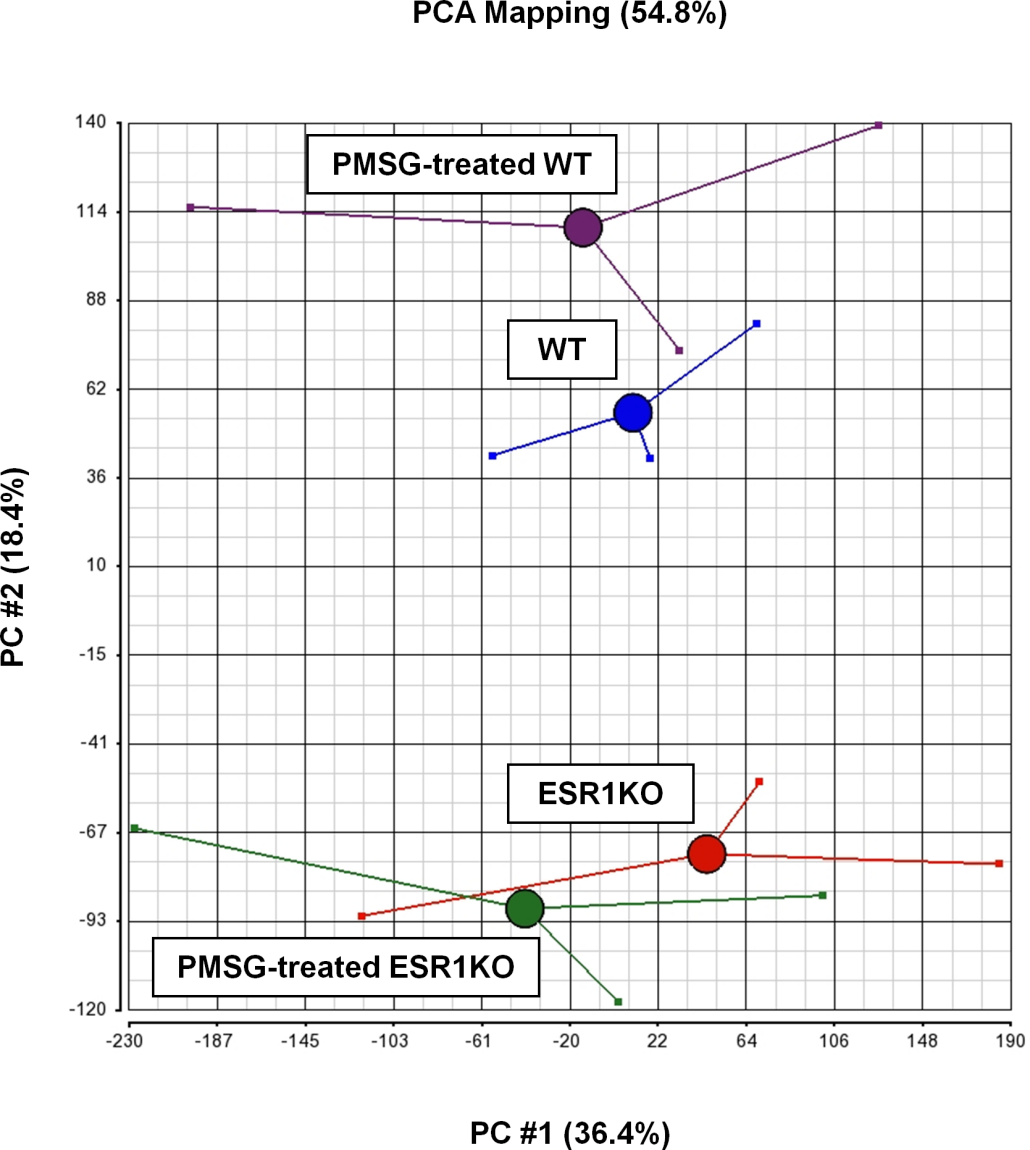
Principal component analysis (PCA) of the microarray-derived transcriptomal results for oviducts collected from ESR1KO mice and WT littermates at 23 days of age, or treated with 5 IU PMSG at 23 days of age and collected 48 h later. Red: ESR1KO, Blue: WT, Green: PMSG-treated ESR1KO, Purple: PMSG-treated WT.

Microarray data were analyzed by importing raw expression intensity values (*.cel files) into Partek Genomics Suite 6.6 (Partek Inc., St. Louis, MO), where the GC-Robust Multiarray Analysis algorithm (GC-RMA), quantile normalization, and Median Polish was applied for GeneChip background correction, log base 2 transformation, conversion of expression values and probeset summarization. Annotation was performed using NetAffx annotation database (Release 34) on December 3^rd^, 2014. Quality of data was assessed using light intensity expression values on a per chip and per gene basis and visualized as box plots (Fig 1). Principal component analysis (PCA) was conducted to determine the quality of the microarray hybridization and visualize the general data variation among the chips (Fig 2, [13]).

### Statistical, Gene Ontology and Pathway Analysis

To detect differentially expressed genes (DEG’s) and the interaction between genotype and PMSG treatment, the normalized and background adjusted microarray data were imported into Partek Genomics suite 6.6 (Partek, Inc.) and a two-way ANOVA performed with factor 1 being genotype and factor 2 being PMSG treatment. Statistical significance of difference for each gene was set to P-value <0.01 with Benjamini-Hochberg multiple testing correction for false discovery rate (FDR) < 0.13. Genes considered significant in the overall model (P < 0.01, FDR < 0.13) were then subjected to pairwise comparisons using Fischer’s Least Significant Difference (LSD) test to estimate the significance of difference for each gene in each comparison. Genes showing a mean difference in signal intensity of at least 2-fold change and a P-value < 0.01 were considered differentially expressed.

With our primary objective of identifying estradiol/ESR1-dependent affects on the transcriptome, the 1185 DEG’s identified in the oviducts of PMSG-treated ESR1KO versus PMSG-treated WT mice was subjected to gene ontology and pathway analysis. Differentially expressed genes were interrogated for their gene ontology (GO) classes using Partek Genomics Suite 6.6 (Partek, Inc.). Partek derives gene ontology classifications from geneontology.org and/or the affymetrix database. GO hierarchies leads to the division of the gene list into significant classifications when the observed number of differentially expressed genes in a GO category is greater than expected. Statistical analysis for significant classifications was performed using Fischer’s exact test, right-tailed. A P-value < 0.01 is suggestive of an over representation of genes from within a particular GO category, indicative of a functional effect.

These same DEG’s (PMSG-treated ESR1KO versus PMSG-treated WT) were then subjected to Ingenuity Pathway Analysis (IPA®, QIAGEN Redwood City, www.qiagen.com/ingenuity) which uses multiple databases to extrapolate significant pathways based on the number of significant genes within our list and known to be involved in a particular pathway. To determine significant pathways in the oviducts of PMSG-treated ESR1KO versus PMSG-treated WT treated mice, a Fischer’s exact test was performed with significance set to P-value < 0.05.

### Real-time RT-PCR

Pathway analysis revealed that the most significant pathways were reflective of immune responses. Therefore, to validate the microarray analysis, real-time RT-PCR was performed to quantify the level of expression of a selection of immune-regulating mRNAs: chemokine (C-C motif) ligand 5, *Ccl5*; cytochrome P450, family 26, subfamily A, polypeptide 1, *Cyp26a*; hematopoetic prostaglandin D synthase, *Hpgds*; interleukin 18 receptor accessory protein, *Il18rap*; prostaglandin-endoperoxide synthase 2, *Ptgs2*; lecithin retinol acyltransferase (phosphatidylcholine-retinol O-acyltransferase), *Lrat*; S100 calcium binding protein A8, *S100a8*; and uroplakin 1A, *Upk1a*. Real-time RT-PCR was performed using an Eppendorf Mastercycler ep *realplex*^2^ system (Eppendorf) using iQ SYBR Green Supermix (Bio-RAD, Hercules, CA), as described before [15,17].

Briefly, cDNA was synthesized using the SuperScript III 1^st^ Strand Synthesis System (Invitrogen), with 0.5 μg of RNA used for each reverse transcription reaction. Real-time RT-PCR was performed with a total volume of 25 μl per reaction, with each reaction containing 5 μL of cDNA, 1 μL of a 10 μm stock of each primer (forward and reverse), 12.5 μL of 2× SYBR Green PCR Master Mix, and 5.5 μL of nuclease-free water. Gene expression was analyzed by the 2−^ΔΔ^CT method [19]. The typical dissociation curves of these cDNA, plus *Gapdh* as the housekeeping gene was confirmed. Oligonucleotide primer pairs (Integrated DNA technologies, Coralville, IA) are described in Table 1.

**Table 1:** Primer sequences (forward and reverse) and PCR product sizes used for real-time RT-PCR analyses.

Name	Accession #	Primer sequence (5'–3')	Product size
*Ccl5*	NM_013653.3	F: CCT CAC CAT ATG GCT CGG AC	121
		R: ACG ACT GCA AGA TTG GAG CA	
*Cyp26a1*	NM_007811.2	F: AGC TCC TGA TTG AGC ACT CG	292
		R: GGA GGA TTC AAT CGC AGG GT	
*Hpgds*	NM_019455.4	F: CAC TAG TTT CCT GGC TAG GGT	383
		R: TGT CAC AGC TCC TTT CCT TGT	
*Il18rap*	NM_010553.3	F: TGC AAT GAA GCG GCA TCT GT	133
		R: CCG GTG ATT CTG TTC AGG CT	
*Lrat*	NM_023624.4	F: GTC GCC CAT CTA ATG CCT GA	324
		R: CTG TGG ACT GAT CCG AGA GC	
*Ptgs2*	NM_011198.4	F: CAT CCC CTT CCT GCG AAG TT	178
		R: CAT GGG AGT TGG GCA GTC AT	
*S100a8*	NM_013650.2	F: CTT TCG TGA CAA TGC CGT CTG	99
		R: AGA GGG CAT GGT GAT TTC CT	
*Upk1a*	NM_026815.2	F: TGA GCA AGA GTG TTG TGG CA	240
		R: CAC GAT ATG CCC CAC GTG TA	
*Gapdh*	GU214026.1	F: CCC CCA ATG TGT CCG TCG TGG	201
		R: TGA GAG CAA TGC CAG CCC CG	

For statistical analysis of real-time RT-PCR results, datasets were first tested for normality and equal variance. When appropriate, data were transformed before statistical analysis. A one-way ANOVA using SigmaStat 3.5 (Systat Software, Inc., Point Richmond, CA, USA) was used to determine differences in levels of mRNA. When differences were detected a Fischer’s Least Significant Difference (LSD) test was used to determine which genes differed.

## Results

### Detection of DEG’s by Microarray Analysis

After chip normalization, a statistical two-way ANOVA and pairwise comparison (LSD test) was performed to generate a list of 2428 differentially expressed genes (P < 0.01, FDR < 0.13). Genotype affected the expression of 2215 genes, PMSG affected the expression of 465 genes, and Genotype x PMSG affected the expression of 438 genes (Table 2). Following removal of unannotated and duplicate probesets, DEG’s were further subdivided between up- and down-regulated genes. The identity of the 20 most highly up- and down-regulated genes in the oviducts of ESR1KO versus WT mice, and PMSG-treated ESR1KO versus PMSG-treated WT mice are provided in Tables 3–6. The identity of all genes determined to be differentially expressed by two-way ANOVA is provided in S1 Table.

**Table 2:** Number of differentially expressed genes (DEG's) identified by microarray analysis and pair-wise comparisons between genotypes and treatments.

Parameter	No. of DEG's		
Model	2428		
Genotype	2215		
PMSG treatment	465		
Genotype by PMSG interaction	438		
**Pairwise comparisons**	**No. of DEG's**	**Up-regulated**	**Down-regulated**
PMSG-treated ESR1KO vs. ESR1KO	37	31 (84%)	6 (16%)
PMSG-treated WT vs. WT	318	164 (52%)	154 (48%)
PMSG-treated ESR1KO vs. PMSG-treated WT	1185	689 (58%)	496 (42%)
ESR1KO vs. WT	664	328 (49%)	336 (51%)

Significance set to P-value < 0.01 with FDR determined from the Benjamini-Hochberg multiple testing correction < 0.13. For pairwise comparisons, unannotated and duplicate probe sets were removed from gene lists, and only genes with at least a 2-fold change in level of expression were considered differentially expressed.

**Table 3:** Top 20 most highly up-regulated mRNAs in the oviducts of ESR1KO versus WT mice. Overall Model: P < 0.01 and at least a 2-fold change in gene expression.

Gene Symbol	Gene Description	P-value	Fold-Change
*Sult1e1*	sulfotransferase family 1E, member 1	< 0.001	33.1834
*Chodl*	chondrolectin	< 0.001	25.6824
*Avpr1a*	arginine vasopressin receptor 1A	< 0.001	24.7639
*Synpr*	synaptoporin	< 0.001	22.5126
*Glb1l3*	galactosidase, beta 1 like 3	< 0.001	21.5821
*BC048679*	cDNA sequence BC048679	< 0.001	21.0171
*Ager*	advanced glycosylation end product-specific receptor	< 0.001	17.8374
*2310043J07Rik*	RIKEN cDNA 2310043J07 gene	< 0.001	15.0383
*Pcdh8*	protocadherin 8	< 0.001	14.7291
*Lemd1*	LEM domain containing 1	< 0.001	14.6033
*Slc47a1*	solute carrier family 47, member 1	< 0.001	14.215
*Adamts16*	a disintegrin-like and metallopeptidase (reprolysin type) with thrombospondin type 1 mo	< 0.001	13.9153
*Tnfrsf21*	tumor necrosis factor receptor superfamily, member 21	< 0.001	12.0567
*9330159F19Rik*	RIKEN cDNA 9330159F19 gene	< 0.001	11.991
*S100a8*	S100 calcium binding protein A8 (calgranulin A)	0.002	11.9115
*Mmp7*	matrix metallopeptidase 7	< 0.001	11.8858
*Kcnd2*	potassium voltage-gated channel, Shal-related family, member 2	< 0.001	11.8108
*AA986860*	expressed sequence AA986860	< 0.001	11.5917
*Cdh16*	cadherin 16	< 0.001	11.5566
*Trank1*	tetratricopeptide repeat and ankyrin repeat containing 1	< 0.001	11.4235

Fold-Change in gene expression and P-Values are indicated. Positive changes in fold-change represent increased expression in the oviducts of ESR1KO mice.

**Table 4:** Top 20 most highly down-regulated mRNAs in the oviducts of ESR1KO versus WT mice. Overall Model: P < 0.01 and at least a 2-fold change in gene expression.

Gene Symbol	Gene Description	P-value	Fold-Change
*Pcdh17*	protocadherin 17	< 0.001	-10.6901
*Csf3*	colony stimulating factor 3 (granulocyte)	0.002	-11.5917
*Tshr*	thyroid stimulating hormone receptor	< 0.001	-11.8455
*Col6a4*	collagen, type VI, alpha 4	< 0.001	-12.206
*Akr1c14*	aldo-keto reductase family 1, member C14	< 0.001	-12.2614
*Upk1a*	uroplakin 1A	0.001	-12.9078
*Slc6a2*	solute carrier family 6 (neurotransmitter transporter, noradrenalin), member 2	< 0.001	-13.1394
*Lrat*	lecithin-retinol acyltransferase (phosphatidylcholine-retinol-O-acyltransferase)	< 0.001	-13.3562
*Ano4*	anoctamin 4	0.002	-13.6892
*Gp1bb*	glycoprotein Ib, beta polypeptide	< 0.001	-15.0412
*Stat5a*	signal transducer and activator of transcription 5A	< 0.001	-16.1053
*Rtn1*	reticulon 1	< 0.001	-18.0311
*Syn2*	synapsin II	< 0.001	-23.8067
*Ramp3*	receptor (calcitonin) activity modifying protein 3	< 0.001	-24.6301
*Mlc1*	megalencephalic leukoencephalopathy with subcortical cysts 1 homolog (human)	< 0.001	-29.3425
*Hpgds*	hematopoietic prostaglandin D synthase	0.001	-32.2313
*Cyp26a1*	cytochrome P450, family 26, subfamily a, polypeptide 1	0.001	-39.5376
*Dcpp1 /// Dcpp2 /// Dcpp3*	demilune cell and parotid protein 1 /// demilune cell and parotid protein 2 /// demilune cell and parotid protein 3	< 0.001	-47.7492
*2300002M23Rik*	RIKEN cDNA 2300002M23 gene	0.003	-71.6374
*Dcpp3*	demilune cell and parotid protein 3	< 0.001	-77.6391

Fold-Change in gene expression and P-Values are indicated. Negative changes in fold-change represent decreased expression in the oviducts of ESR1KO mice.

**Table 5:** Top 20 most highly up-regulated mRNAs in the oviducts of PMSG-treated ESR1KO versus PMSG-treated WT mice. Overall Model: P < 0.01 and at least a 2 fold-change in gene expression.

Gene Symbol	Gene Description	P-value	Fold-Change
*BC048679*	cDNA sequence BC048679	<0.001	222.15
*Apod*	apolipoprotein D	<0.001	98.02
*Cdh16*	cadherin 16	<0.001	50.13
*Chodl*	Chondrolectin	<0.001	48.14
*Sult1e1*	sulfotransferase family 1E, member 1	<0.001	46.32
*G6pc2*	glucose-6-phosphatase, catalytic, 2	<0.001	42.88
*Pla2g10*	phospholipase A2, group X	<0.001	40.8
*Fgf18*	fibroblast growth factor 18	<0.001	38.17
*Avpr1a*	arginine vasopressin receptor 1A	<0.001	35.78
*Aldh1a3*	aldehyde dehydrogenase family 1, subfamily A3	<0.001	34.76
*Lrrtm1*	leucine rich repeat transmembrane neuronal 1	<0.001	29.44
*Serpina1b*	serine (or cysteine) preptidase inhibitor, clade A, member 1B	<0.001	27.57
*Ager*	advanced glycosylation end product-specific receptor	<0.001	22.77
*Synpr*	Synaptoporin	<0.001	22.32
*S100a8*	S100 calcium binding protein A8 (calgranulin A)	<0.001	20.77
*Adamts16*	a disintegrin-like and metallopeptidase (reprolysin type) with thrombospondin type 1 motif 16	<0.001	19.82
*Il18r1*	interleukin 18 receptor 1	<0.001	19.45
*S100a9*	S100 calcium binding protein A9 (calgranulin B)	0.0015	17.86
*Atp6v1b1*	ATPase, H+ transporting, lysosomal V1 subunit B1	<0.001	17.8
*Wnt7a*	wingless-related MMTV integration site 7A	<0.001	17.49

Fold-Change in gene expression and P-Values are indicated. Positive changes in fold-change represent increased expression in the oviducts of PMSG-treated ESR1KO mice.

**Table 6:** Top 20 most highly down-regulated mRNAs in the oviducts of PMSG-treated ESR1KO versus PMSG-treated WT mice. Overall Model: P < 0.01 and at least a 2 fold-change in gene expression.

Gene Symbol	Gene Description	P-value	Fold-Change
*Dcpp3*	demilune cell and parotid protein 3	<0.001	-770.92
*2300002M23Rik*	RIKEN cDNA 2300002M23 gene	<0.001	-524.94
*Cyp26a1*	cytochrome P450, family 26, subfamily a, polypeptide 1	<0.001	-131.13
*Tshr*	thyroid stimulating hormone receptor	<0.001	-121.71
*Dcpp1/2/3*	demilune cell and parotid protein 1/demilune cell and parotid protein 2/demilune cell and parotid protein 3	<0.001	-106.94
*Syn2*	synapsin II	<0.001	-91.45
*Slc6a2*	solute carrier family 6 (neurotransmitter transporter, noradrenalin), member 2	<0.001	-77.2
*Upk1a*	uroplakin 1A	<0.001	-77.14
*Hpgds*	hematopoietic prostaglandin D synthase	<0.001	-73.37
*Klk1b24*	kallikrein 1-related peptidase b24	<0.001	-73.19
*Greb1*	gene regulated by estrogen in breast cancer protein	<0.001	-64.6
*Klk1b1*	kallikrein 1-related peptidase b1	<0.001	-62.94
*Klk1b21*	kallikrein 1-related peptidase b21	<0.001	-53.58
*Lrat*	lecithin-retinol acyltransferase (phosphatidylcholine-retinol-O-acyltransferase)	<0.001	-53.53
*Gria1*	glutamate receptor, ionotropic, AMPA1 (alpha 1)	<0.001	-46.45
*Akr1c14*	aldo-keto reductase family 1, member C14	<0.001	-45.65
*Stat5a*	signal transducer and activator of transcription 5A	<0.001	-44.22
*Col6a4*	collagen, type VI, alpha 4	<0.001	-43.1
*Rasd1*	RAS, dexamethasone-induced 1	<0.001	-42.74
*Adh7*	alcohol dehydrogenase 7 (class IV), mu or sigma polypeptide	<0.001	-42.46

Fold-Change in gene expression and P-Values are indicated. Negative changes in fold-change represent decreased expression in the oviducts of PMSG-treated ESR1KO mice.

### Verification of selected DEG’s

The expression of mRNA for *Ccl5*, *Cyp26a1*, *Hpgds*, *Il18rap*, *Lrat*, *Ptgs2*, *S100a8*, and *Upk1a* in the oviducts of PMSG-treated ESR1KO versus PMSG-treated WT mice was determined by real-time RT-PCR. A comparison of the results obtained by real-time RT-PCR and microarray analysis is presented in Table 7 as a validation of the microarray platform. Overall, real-time RT-PCR revealed the same directional trends in gene expression that were observed by the microarray analysis.

**Table 7:** Comparison of gene expression for selected mRNAs by microarray and real-time RT-PCR in the oviducts of PMSG-treated ESR1KO versus PMSG-treated WT mice.

	Microarray		Real-time RT-PCR	
Gene Symbol	Fold-Change	P-Value	Fold-Change	P-Value
*Ccl5*	2	0.166	4.2	< 0.001
*Cyp26a1*	-131.1	< 0.001	-27.4	< 0.001
*Hpgds*	-73.4	< 0.001	-13.4	< 0.001
*Il18rap*	1	0.22	6.2	< 0.001
*Lrat*	-53.5	< 0.001	-21.8	< 0.001
*Ptgs2*	1.57	0.24	2.19	< 0.001
*S100a8*	20.7	0.0006	18.02	< 0.001
*Upk1a*	-77.1	< 0.001	-38.9	< 0.001

Fold-Change in gene expression and P-Values are indicated after analysis by microarray and by independent real-time RT-PCR. Positive changes in fold change represent increased expression in the oviducts of PMSG-treated ESR1KO mice.

### Gene Ontology Analysis of DEG’s in the oviducts of PMSG-treated ESR1KO versus PMSG-treated WT mice

**Fig 3:**
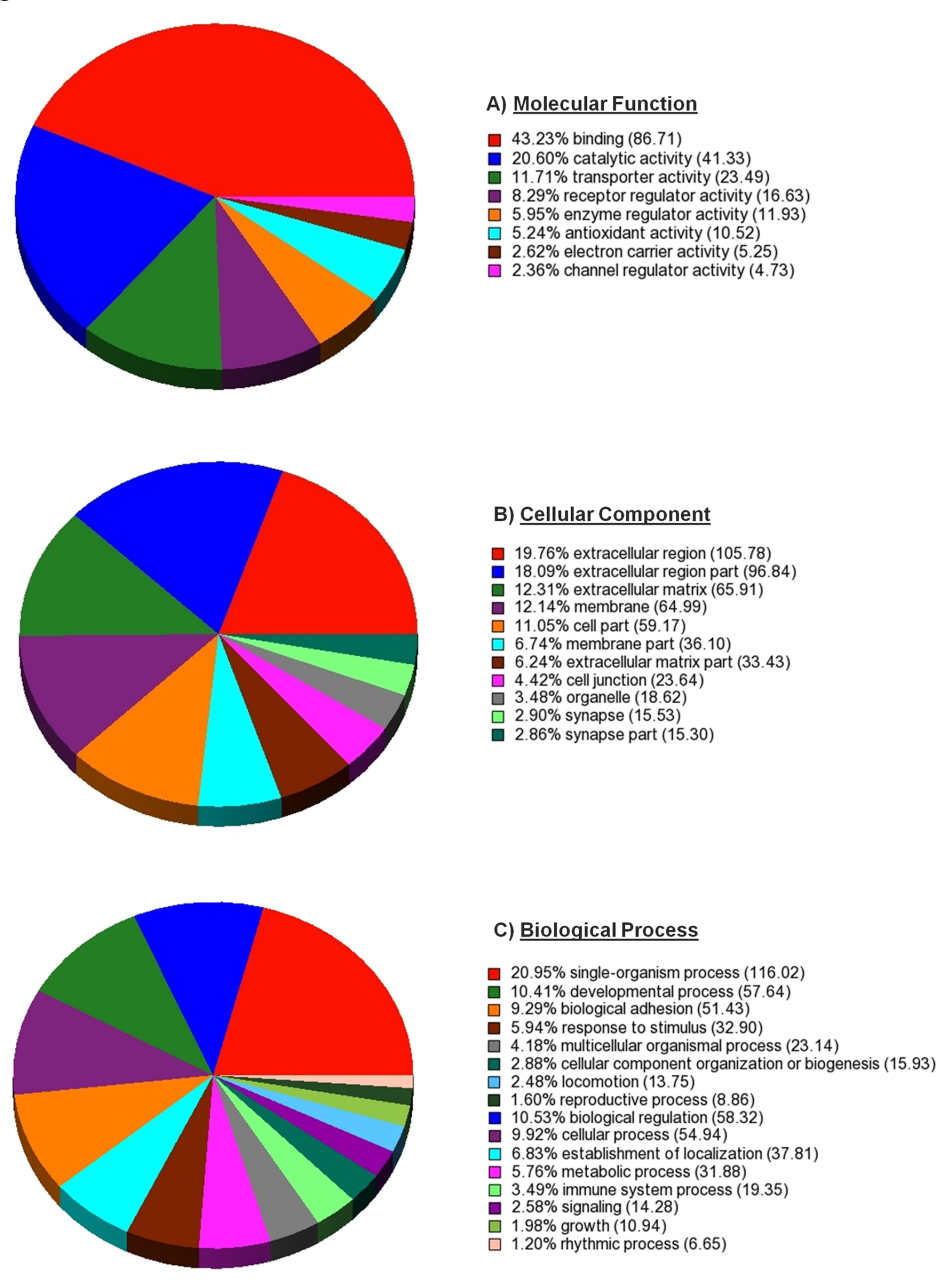
Gene Ontology (GO) analysis with Molecular Function, Cellular Component and Biological Processes categories. Pie chart shows the distribution of the DEG’s in the oviducts of PMSG-treated ESR1KO versus PMSG-treated WT mice that were matched to A) a Molecular Function, B) a Cellular Component, and C) a Biological Process, using GO.

Consistent with our overall goal of identifying estradiol/ESR1-dependent affects on the oviduct, the molecular functions, cellular components, and biological processes of DEG’s expressed in the oviducts of PMSG-treated ESR1KO versus PMSG-treated WT mice were determined by Gene Ontology (GO) Analyses with significance set to enrichment P-value < 0.01. The significantly enriched molecular function categories using GO are shown in Fig 3A. The categories with the highest enrichment score within molecular functions were binding, catalytic and transporter activities. Significantly enriched cellular component categories are shown in Fig 3B. The most highly enriched cellular component categories were the extracellular region/matrix and the cell membrane. Significantly enriched biological processes are indicated in Fig 3C, with the most highly enriched categories including those involving a single organism, modulating a measurable attribute (biological regulation) and specific outcome (developmental process).

### Ingenuity Pathway Analysis of DEG’s in the oviducts of PMSG-treated ESR1KO versus PMSG-treated WT mice

**Fig 4:**
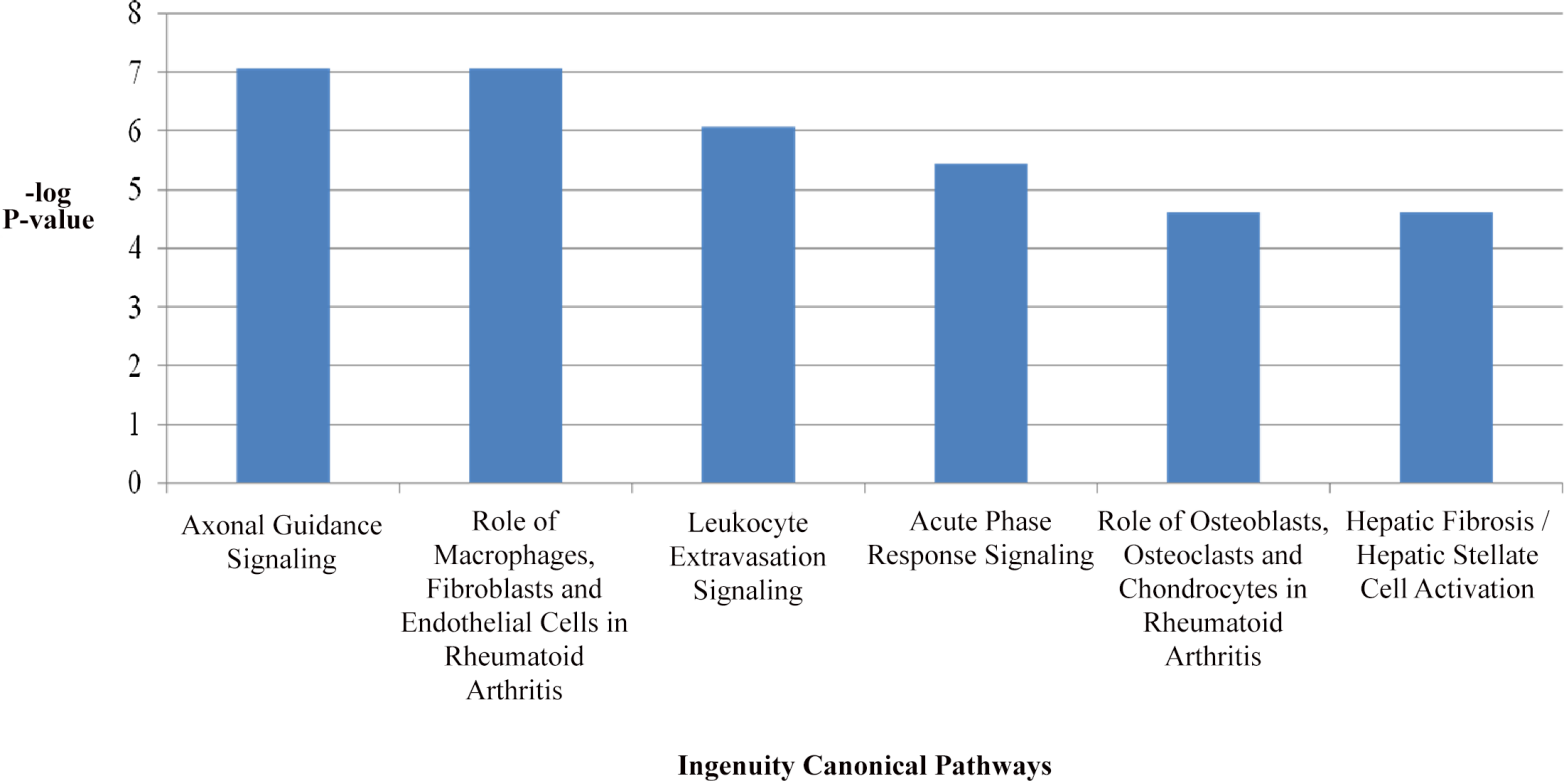
Most highly significant canonical pathways identified in the oviducts of PMSG-treated ESR1KO versus PMSG-treated WT identified using QIAGEN’S Ingenuity Pathway Analysis.

Canonical pathway analysis of DEG’s from PMSG-treated ESR1KO versus PMSG-treated WT mice was performed using QIAGEN’S Ingenuity Pathway Analysis (IPA, QIAGEN, Redwood City, www.qiagen.com/ingenuity). The six most significant pathways identified by Ingenuity Pathway Analysis software are provided in Fig 4, and are reflective of ESR1-dependent regulation of the immune response. The top upstream regulators were tumor necrosis factor (TNF), interferon gamma (IFNG), interleukin 1β (IL1B), amyloid β (A4) precursor protein (APP) and interleukin 13 (IL13). The top regulator effect networks included a disintegrin-like and metallopeptidase (reprolysin type) with thrombospondin type 1 (ADAMTS12), homeodomain interacting protein kinase 2 (HIPK2), interleukin 22 (IL22), interleukin 27 (IL27), toll-like receptor 3 (TLR3), toll-like receptor 4 (TLR4) and conserved helix-loop-helix ubiquitous kinase (CHUK) as their primary regulators. With pathway analysis indicating the immune response as a primary canonical pathway, a listing of differentially expressed mRNAs specifically encoding chemokines, interleukins and their receptors in the oviducts of PMSG-treated ESR1KO versus PMSG-treated WT mice is provided as Table 8. All the significant pathways (P-value < 0.05) and the corresponding molecules differentially expressed within the pathways from PMSG-treated ESR1KO versus PMSG-treated WT mice are provided in S2 Table.

**Table 8:** Differentially expressed mRNAs encoding chemokines, interleukins and their receptors in the oviducts of PMSG-treated ESR1KO versus PMSG-treated WT mice. Overall Model: P < 0.01 and at least a 2-fold change in gene expression.

Gene Symbol	Gene Description	P-value	Fold-Change
*Cx3cl1*	chemokine (C-X3-C motif) ligand 1	0.004	2.25
*Cxcl12*	chemokine (C-X-C motif) ligand 12	< 0.001	4.21
*Cxcl14*	chemokine (C-X-C motif) ligand 14	0.001	-3.31
*Cxcl16*	chemokine (C-X-C motif) ligand 16	0.011	1.73
*Cxcl17*	chemokine (C-X-C motif) ligand 17	0.003	3.68
*Cxcr4*	chemokine (C-X-C motif) receptor 4	0.063	1.61
*Cxcr7*	chemokine (C-X-C motif) receptor 7	< 0.001	-3.32
*Il13ra2*	interleukin 13 receptor, alpha 2	0.003	4.48
*Il15*	interleukin 15	< 0.001	2.70
*Il15ra*	interleukin 15 receptor, alpha chain	< 0.001	5.16
*Il16*	interleukin 16	0.002	1.62
*Il17ra*	interleukin 17 receptor A	< 0.001	-2.25
*Il17rb*	interleukin 17 receptor B	< 0.001	14.38
*Il17re*	interleukin 17 receptor E	0.002	3.00
*Il18*	interleukin 18	0.002	1.82
*Il18bp*	interleukin 18 binding protein	< 0.001	-20.01
*Il18r1*	interleukin 18 receptor 1	< 0.001	19.45
*Il1r1*	interleukin 1 receptor, type I	0.001	2.14
*Il1rap*	interleukin 1 receptor accessory protein	< 0.001	1.53
*Il33*	interleukin 33	< 0.001	11.69
*Il7*	interleukin 7	0.001	3.94
*Ilf2*	interleukin enhancer binding factor 2	0.012	1.31

Fold-Change in gene expression and P-Values are indicated. Positive changes in fold-change represent increased expression in the oviducts of PMSG-treated ESR1KO mice.

## Discussion

The objective of this study was to determine estradiol/ESR1-dependent changes to the transcriptome of the mouse oviduct, with the overall goals of increasing our understanding of steroidal regulation of this often overlooked reproductive organ, and to provide the identity of ESR1-regulated genes that may prove to be important modulators of oviductal function and fertility in the future. While our focus was on the identification and bioinformatic analysis of DEG’s in the oviducts of PMSG-treated WT versus PMSG-treated ESR1KO mice, the identity of all DEG’s identified by this analysis have been provided (S1 Table), and the raw data (*.cel files) plus the GC-RMA-normalized and log_2_ transformed transcript data have been deposited into the Gene Expression Omnibus. Important to note, whole oviducts were collected for transcriptomal analysis from WT and ESR1KO mice. Future research of targeted mRNAs identified by this analysis will therefore need to include determination of potential differences in spatial location of a gene or protein between the ampulla and isthmus, as well as cellular localization within a specific section of the oviduct. Furthermore, this study was performed to determine estradiol/ESR1-dependent regulation; genomic signaling via ESR2 and non-genomic effects of estradiol on the oviduct via activation of G-protein-coupled receptor 30 (GPR30, [20]) should not be overlooked, nor potential interactions. Indeed, regardless of treatment with PMSG, we observed that ablation of ESR1 resulted in a 1.6-fold increase in the expression of mRNA for *Gpr30* in the oviduct (S1 Table).

ESR1-dependent regulation of immune function was a leading canonical pathway identified. Of note, inflammation and the immune response is a required physiological occurrence within the oviduct as this organ is exposed to freshly ovulated cumulus-oocyte complexes, associated follicular debris, spermatozoa, seminal fluids and possibly an array of foreign pathogens at ovulation and/or mating [21,22,23,24]. However, salpingitis or aberrant inflammation is also one of the most common forms of pelvic inflammatory disease (PID) and is one of the most important components of the PID spectrum due to its impact on female fertility (reviewed in [25]). This uncontrolled inflammation results in oviductal epithelial cell death, tubal scarring and eventually occlusion [26,27,28,29,30], making identification of the specific transcripts involved in ESR1-dependent regulation of immune function a salient finding of this transcriptomal analysis.

Overall, with significance set to P < 0.01, greater than two thousand transcripts were determined to be differentially regulated. A pairwise comparison of DEG’s in 23 day old ESR1KO and WT mice (i.e. without PMSG-stimulated production of ovarian estradiol) revealed 664 DEG’s, and a pairwise comparison of DEG’s in PMSG-treated WT versus PMSG-treated ESR1KO oviducts revealed 1185 differentially regulated genes, which were subsequently analyzed for gene ontology as well as with Ingenuity Pathway Analysis (IPA®, QIAGEN), which uses multiple databases to extrapolate significant canonical pathways based on the number of genes expected to be expressed within each pathway. With the exception of axonal guidance signaling (reviewed in [31]), the other top canonical pathways (Fig 4) were all directly related to immune function, as were the 5 top upstream regulators (TNF, IFNG, IL1B, APP and IL13).

We have previously reported that the expression of the hematopoetic form of prostaglandin D synthase (HPGDS), a putative regulator of inflammation in the oviduct, is dependent upon ESR1 [15]. In that study, genetic deletion of ESR1 reduced the expression of mRNA encoding *Hpgds* and inhibition of HPGDS in wild-type mice by treatment with HQL-79 (Cayman Chemical, Ann Arbor, MI) resulted in a 2.3-fold increase in the expression of mRNA for one of the upstream regulators identified herein (IL13), a 2.9-fold increase in the expression of mRNA for chemokine (C-X-C motif) ligand 12 (*Cxcl12)*, as well as a 1.8-fold increase in the expression of mRNA for TNF receptor superfamily, member 1b (*Tnfrsf1b*) which is also known as TNF receptor 2 (TNFR2), one of the two receptors that bind TNFα. Herein, the expression of mRNA for *Cxcl12* and *Tnfrsf1b* was 4.2- and 2.5-fold higher in the oviducts of PMSG-treated ESR1KO versus PMSG-treated WT mice (Table 8 and S1 Table). Taken together, our results are consistent with regulation of inflammation within the oviduct acting, in part, through ESR1-dependent HPGDS signaling. Of physiological relevance, the expression of TNFα is reported to increase after infection of human oviducts with *Neisseria gonorrhoeae in vitro* [32] and genetic deletion of IL13 in mice improves the rate of clearance after genital infection with *Chlamydia muridarum* [33], two bacterial pathogens known to induce an inflammatory response within the oviduct [34,35]. Interestingly, the expression of mRNA encoding IL13 receptor, alpha 2 (*Il13ra2*), but not *Il13*, was increased in the oviducts of PMSG-treated ESR1KO versus PMSG-treated WT mice (Table 8).

Of the mRNAs selected for independent analysis by real-time RT-PCR, directional trends were consistent among microarray and RT-PCR analyses (Table 7). Analysis by real-time RT-PCR also revealed that the expression of *Ptgs2* and *Il18rap* was increased in the oviducts of PMSG-treated ESR1KO versus PMSG-treated WT mice. Importantly, for these two transcripts, microarray analysis revealed the same directional trend and a similar magnitude or fold-change in expression. Estradiol is a known regulator of PTGS2 in the oviduct [36], and IL18RAP together with the receptor IL18R1 (interleukin 18 receptor 1) mediates IL18-dependent activation [37,38]. IL18 is a Caspase-1-dependent inflammatory cytokine induced by infection with *C*. *trachomatis* [39]. We observed a 19-fold increase in the expression of *Il18r1* in the oviducts of PMSG-treated ESR1KO versus PMSG-treated WT mice (Tables 5–8), which is also consistent with the regulation of the IL18 receptor by estradiol, as reported in the uterine endometrium [40]. Among the other DEG’s encoding interleukins, mRNA encoding IL17 receptors A, B and E (*Il17ra*, *Il17rb* and *Il17re*) as well as interleukin 1 receptor, type 1 (*Il1r1*) and interleukin 1 receptor accessory protein (*Il1rap*) differed in the oviducts of PMSG-treated ESR1KO versus PMSG-treated WT mice (Table 8). Mice deficient in IL17 display an attenuated response to genital infection with *C*. *muridarium* [41], whereas IL1 is an established regulator of *C*. *trachomatis*-induced inflammation in the oviduct [42]. Our results therefore revealing estradiol/ESR1-dependent changes within the oviduct of transcripts reported to affect inflammation in response to targeted bacterial challenges by others.

Differences in the magnitude of change were observed for some transcripts by microarray analysis versus real-time RT-PCR, as expected [43], whereas technique did not affect the magnitude of change for others. For example, microarray analysis revealed that the S100 calcium binding proteins A8 and A9 were increased by 21-and 18-fold, respectively, in PMSG-treated ESR1KO versus PMSG-treated WT oviducts (Table 5). Real-time RT-PCR confirmed the increased expression of S100A8, with the relative expression for this mRNA increased by 18-fold in the PMSG-treated ESR1KO oviduct (Table 7). The S100 calcium binding proteins are pro-inflammatory, inducing chemotaxis and adhesion of neutrophils [44] and increasing IL1β secretion by IFNG-primed monocytes [45]. Again, consistency with targeted bacterial studies is observed; IL1 signaling and IL1β secretion are involved in the response of the oviduct to infection with *C*. *muridarium* [46].

To conclude, this transcriptomal analysis can provide us with great insight into estradiol/ESR1-dependent regulation of oviductal gene expression and presumably function. Pathway analysis illustrated the complex role of estradiol and ESR1 in regulating oviductal function and identified putative ESR1-dependent molecules involved. This dataset can now be examined in greater detail by others with the hope of expanding our understanding of ESR1-dependent regulation of physiological function in this key reproductive organ.

## Supporting Information

S1 Table Listing of the 2428 differentially expressed genes (P < 0.01, FDR < 0.13) identified by two-way ANOVA and pairwise comparison (LSD test). Gene symbol, gene title and all pair-wise comparisons, P-values and fold changes in expression are indicated.
(XLS)

S2 Table Complete results of the canonical pathway analysis of DEG’s from PMSG-treated ESR1KO versus PMSG-treated WT mice, performed using QIAGEN’S Ingenuity Pathway Analysis (IPA, QIAGEN, Redwood City, www.qiagen.com/ingenuity). All the significant pathways (P-value ≤ 0.05) and the corresponding molecules differentially expressed within the pathways from PMSG-treated ESR1KO versus PMSG-treated WT mice are indicated.
(XLS)
